# Primary anorectal malignant melanoma with synchronous hepatic metastases in middle-aged patient: a case report

**DOI:** 10.1093/jscr/rjag654

**Published:** 2026-07-29

**Authors:** Majed Khaled Alrudayni, Nadir Ali Hilal, Rayan Abdullah Almurais, Adi Suliman Aldhali, Abdullah Talea Almutairi, Nouf Yahya Alyousef

**Affiliations:** Colorectal Consultant, Department of General Surgery, King Fahad Specialist Hospital, Buraydah, Qassim 51461, Saudi Arabia; Colorectal Consultant, Department of General Surgery, King Fahad Specialist Hospital, Buraydah, Qassim 51461, Saudi Arabia; General Surgery Specialist, Department of General Surgery, King Fahad Specialist Hospital, Buraydah, Qassim 51461, Saudi Arabia; General Surgery Specialist, Department of General Surgery, Military Hospital Buraydah, Qassim 52558, Saudi Arabia; General Surgery Resident, Department of General Surgery, King Fahad Specialist Hospital, Buraydah, Qassim 51461, Saudi Arabia; Medical Student, University of Tabuk, Faculty of Medicine Tabuk, Tabuk 71491, Saudi Arabia

**Keywords:** anorectal melanoma, malignancy, endoscopic intervention, HMB-45, anal canal

## Abstract

Anorectal malignant melanoma is a rare, notably aggressive malignancy, comprising fewer than 1% of all melanoma cases. Its early symptoms frequently resemble benign anorectal conditions, which may lead to diagnostic delays and poor outcomes. We report a case of a 42-year-old, previously healthy male who presented with a two-month history of progressive constipation, unintentional weight loss, and lower abdominal discomfort associated with melena. Imaging revealed a mass extending from the anal canal into the distal rectum, with hepatic metastases. Histopathological examination confirmed malignant melanoma with positive immunohistochemical staining for HMB-45 and Melan-A, and negative S-100. A diverting functional colostomy was performed for impending obstruction, and first-line nivolumab was initiated, but the patient deteriorated rapidly and died on best supportive care. This case highlights the silent nature of this malignancy and the importance of considering rare yet aggressive tumors when dealing with atypical or unexplained anorectal lesions.

## Introduction

Rectal melanoma is a rare yet notably aggressive malignancy that arises from melanocytes in the mucosa of the anal canal or distal rectum. While most melanomas are cutaneous, malignant mucosal types account for less than 1% of all melanoma cases, with anorectal malignant melanoma (ARMM) representing approximately 0.5% of anorectal cancers [1, 2]. Due to its rarity and vague symptomatology, rectal melanoma is often misdiagnosed or identified at an advanced stage. Common presenting symptoms, including rectal bleeding, changes in bowel habits, or perianal discomfort, are frequently mistaken for benign anorectal disorders, including fissures or hemorrhoids [1–3]. ARMM tends to spread rapidly, and at the time of diagnosis, regional or distant metastases are often already present, most commonly involving the liver, lungs, or lymph nodes [4, 5]. The prognosis remains poor, with a median survival estimate of 12 months, even with combined treatment modalities [2]. Although ARMM has been discussed in international literature, reports remain scarce in Saudi Arabia. We, herein, aim to highlight the silent nature of this malignancy and the importance of considering rare yet aggressive tumors when dealing with atypical or unexplained anorectal symptoms.

## Case presentation

A 42-year-old previously healthy non-Saudi male presented to the outpatient clinic at Buraydah Central Hospital with a two-month history of progressive constipation, early satiety, unintentional weight loss, occasional tenesmus, and lower abdominal discomfort associated with melena for the past 7 days. He denied any overt rectal bleeding and had no relevant past medical, surgical, or family history. On examination, he was cachectic but alert and oriented with stable vital signs. The abdomen was soft with right upper quadrant swelling and tenderness; a digital rectal examination revealed a firm, irregular, pigmented, fungating mass at the 12–1 o’clock position, 0.5 cm from the anal verge, almost completely obstructing the rectal lumen. Initial investigations showed a hemoglobin level of 7.3 g/dL, a white blood cell count of 16 × 10^9^/L, a platelets 278 × 10^9^/L, ALP 142 IU/L, and a LDH 385 IU/L; renal function was normal. Contrast-enhanced computed tomography (CT) of the abdomen and pelvis demonstrated a 5-cm, hyper-enhancing, fungating mass at the left rectal wall extending 5 cm from the anal verge (Fig. 1A), with perirectal fat stranding and meso-rectal fascial involvement (Fig. 1B). Enlarged meso-rectal and bilateral iliac lymph nodes were present (largest 2.2 cm), together with multiple hypodense hepatic lesions (largest 4.6 × 3.6 cm), consistent with hepatic metastases (Fig. 2A and B). Chest imaging was unremarkable. Colonoscopy confirmed an ulcerated, pigmented anorectal mass extending from the anal canal into the lower rectum with near-complete luminal obstruction. Histopathology revealed malignant melanoma; immunohistochemistry was positive for HMB-45 and Melan-A, and negative for S-100, CK AE1/AE3, CK20, LCA, SATB2, DOG1, CD31, synaptophysin, and desmin (Fig. 3A–C). The patient was ECOG performance status 2. A functional colostomy had been performed at King Fahad Specialist Hospital prior to referral to manage the luminal obstruction. Given the advanced stage and the unavailability of BRAF mutation testing, he was commenced on nivolumab 240 mg IV every two weeks. As his condition deteriorated and systemic therapy was no longer feasible, he was transitioned to palliative care and subsequently died.

**Figure 1:**
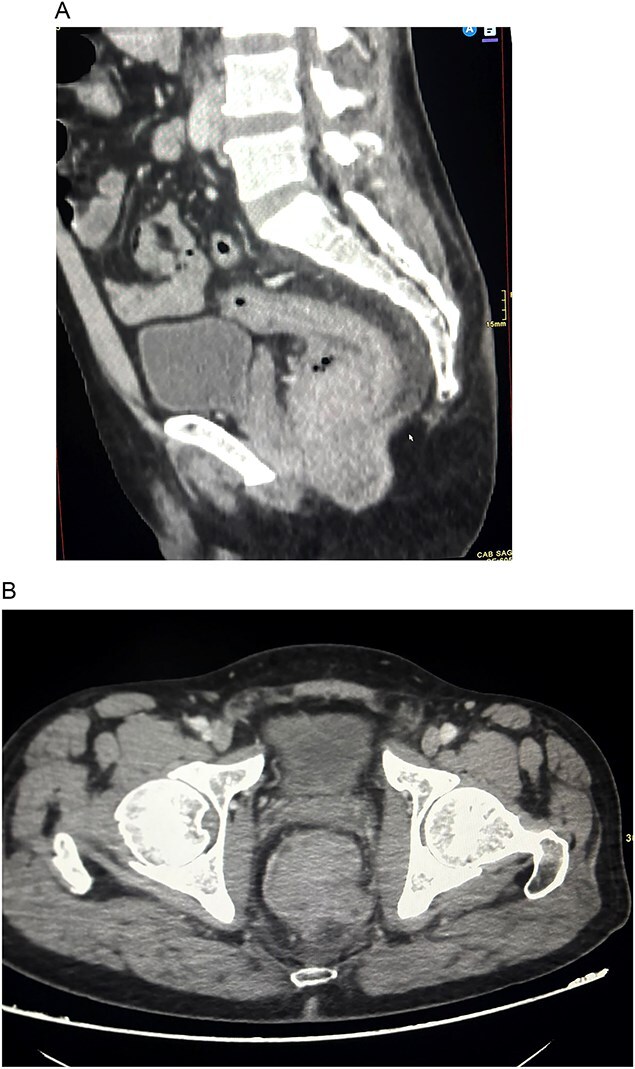
Contrast-enhanced CT of the pelvis demonstrating ARMM. (A) Sagittal CT image showing a large hyperenhancing fungating anorectal mass arising from the distal rectum and anal canal, causing near-complete luminal obstruction with associated rectal wall thickening and local soft tissue infiltration. (B) Axial CT image demonstrating circumferential anorectal wall thickening with intraluminal soft tissue mass and surrounding mesorectal involvement.

**Figure 2:**
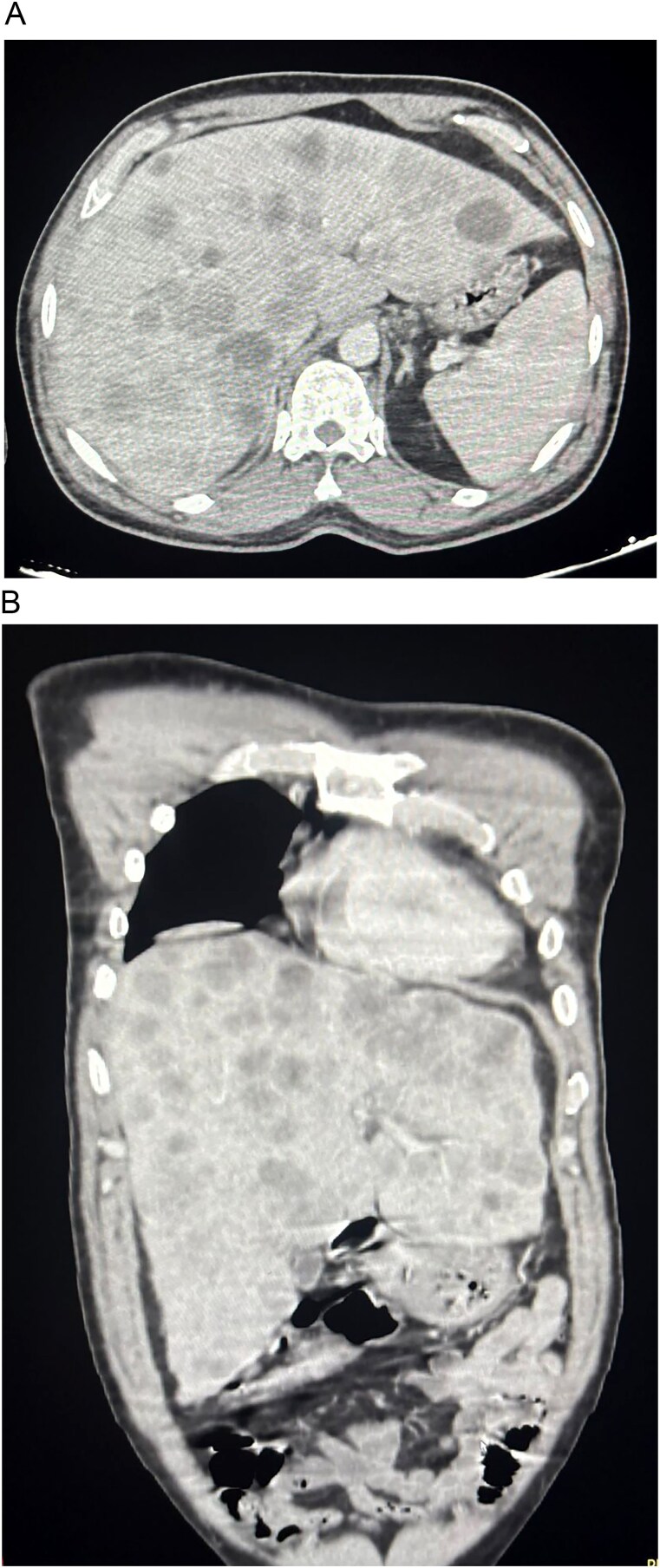
Contrast-enhanced CT demonstrating metastatic disease involving the liver. (A) Axial CT image of the upper abdomen showing multiple hypodense hepatic lesions scattered throughout both hepatic lobes, consistent with metastatic deposits. (B) Coronal CT reconstruction illustrating extensive bilobar hepatic metastases associated with advanced ARMM.

**Figure 3:**
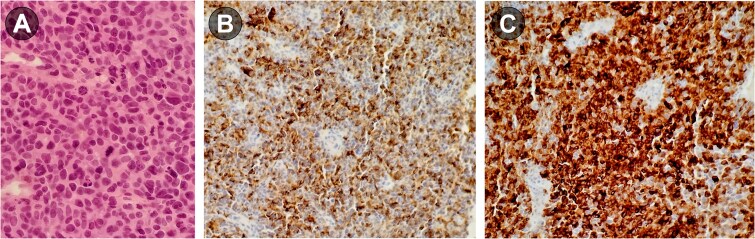
(A) Hematoxylin and eosin staining demonstrating sheets of pleomorphic malignant tumor cells with hyperchromatic nuclei, prominent nucleoli, and frequent mitotic figures, consistent with malignant melanoma. (B) Immunohistochemical staining confirming melanocytic differentiation; tumor cells showing diffuse cytoplasmic positivity for HMB-45 immunostaining. (C) Strong diffuse positivity for Melan-A immunostaining, supporting the diagnosis of ARMM.

## Discussion

ARMM typically presents in patients in their sixties or seventies, and is uncommon in younger adults, though isolated reports have documented the disease in patients as young as 19 [2, 3, 6]. Our patient was a 42-year-old man whose short history of abdominal discomfort, difficulty defecating, and unintentional weight loss progressed rapidly to systemic decline—a clinical trajectory that illustrates both the aggressive biology of ARMM and its tendency toward poor prognosis even in younger, otherwise healthy individuals. Part of what makes ARMM so dangerous is its capacity to mimic other anal and rectal tumors histologically, which delays definitive recognition [2, 7]. In our case, the lesion was initially regarded as a non-specific rectal mass, and melanoma was confirmed only after biopsy—a pattern consistent with reports in which the diagnosis is established only after partial excision or abdominoperineal resection [8–10]. The diagnostic delay was compounded by an atypical clinical presentation; unlike most published cases, which feature a visible anal mass or overt rectal bleeding [8]. Our patient had no anorectal bleeding and instead presented with constitutional and obstructive symptoms. A similar obstructive picture was described by Dnyanmote *et al*. [10] in whose patient ARMM was recognized only after the development of a large-bowel obstruction. Together, these cases underscore that the absence of bleeding does not exclude the diagnosis. By the time imaging was obtained, the disease had already disseminated. The burden of synchronous hepatic metastases precluded curative resection, and a functional colostomy was performed to manage impending obstruction; primary tumor resection was not undertaken. This stands in contrast to the management trajectory of most reported cases, in which complete surgical resection is followed by immunotherapy or radiation [11]. In our patient, deterioration outpaced any meaningful response to nivolumab. While wide local excision and abdominoperineal resection remain the mainstays of locoregional control in the literature, the aggressive biology and rapid systemic progression of ARMM often render these procedures palliative rather than curative—and in the presence of established distant metastases, less morbid local control combined with systemic therapy is increasingly favored [11]. Equally striking was the pace of decline; deterioration in our patient was notably swift, leaving little therapeutic window. This experience reinforces the contemporary emphasis on individualized treatment when curative surgery is not feasible and highlights how narrow the margin can be between potentially curable and uniformly fatal presentations of this disease.

## Conclusion

ARMM remains a rare but aggressive malignancy whose non-specific presentation continues to delay diagnosis, particularly in younger patients. A high index of suspicion across all adult age groups, with a prompt biopsy of any pigmented or fungating anorectal lesion even in the absence of bleeding, offers the single best opportunity to shift this disease from a palliative to a potentially curable diagnosis.
